# NEIGHBORHOOD STRESSORS AND EPIGENETIC AGING AMONG OLDER ADULTS IN THE U.S

**DOI:** 10.1093/geroni/igad104.3495

**Published:** 2023-12-21

**Authors:** Eunyoung Choi, Jennifer Ailshire

**Affiliations:** University of Southern California, Los Angeles, California, United States; University of Southern California, Los Angeles, California, United States

## Abstract

Exposure to stressful neighborhood environments is a well-established risk factor for health deterioration and premature death. However, the underlying biological underpinnings driving these associations are not fully understood. Epigenetics may function as a key molecular pathway to adverse health outcomes among residents of high-stress neighborhoods. This study examines the association of neighborhood stressors (i.e., socioeconomic deprivation, disorder, and low social cohesion) with three epigenetic aging measures (i.e., DunedinPACE and Principal component adjusted (PC) PCPhenoAge and PCGrimAge) based on DNA methylation levels. Further, we identify sub-populations that are particularly vulnerable to the detrimental effects of neighborhood stressors. Data were from the 2016 Health and Retirement Study (HRS) DNA Methylation Sample, merged with neighborhood stressors measured in the HRS 2012/2014 and the 2012-2016 American Community Survey (N=3,201). Multilevel regression models adjusting for confounders showed that perceived high disorder was associated with accelerated DNAm aging for DunedinPACE (β=0.13, 95%CI=0.04-0.23). Perceived low cohesion was associated with accelerated DNAm aging for DunedinPACE (β=0.21, 95%CI=0.08-0.35) and PCGrimAge (β=0.09, 95%CI=0.04-0.14). High deprivation did not have any significant associations. In models of interactions, associations of low cohesion were more pronounced for females in DunedinPACE (β=0.36, 95%CI=0.13-0.60) and PCPhenoAge (β=0.21, 95%CI=0.07-0.35). No other interaction effects were found for race/ethnicity, living arrangement, and education. Our findings indicate that neighborhood stressors can speed up epigenetic aging, with older females being particularly vulnerable to the effects of low cohesion. This study provides insights into the biological foundations of health disparities rooted in neighborhood environments.

